# On How Fas Apoptosis-Independent Pathways Drive T Cell Hyperproliferation and Lymphadenopathy in *lpr* Mice

**DOI:** 10.3389/fimmu.2017.00237

**Published:** 2017-03-10

**Authors:** Dimitrios Balomenos, Rahman Shokri, Lidia Daszkiewicz, Cristina Vázquez-Mateo, Carlos Martínez-A

**Affiliations:** ^1^Department of Immunology and Oncology, Centro Nacional de Biotecnología (CNB-CSIC), UAM Campus de Cantoblanco, Madrid, Spain

**Keywords:** Fas, p21, memory T cells, hyperproliferation, double-negative T cells, lupus autoimmunity, alternative functions, hyperactivation

## Abstract

Fas induces massive apoptosis in T cells after repeated *in vitro* T cell receptor (TCR) stimulation and is critical for lymphocyte homeostasis in Fas-deficient (*lpr*) mice. Although the *in vitro* Fas apoptotic mechanism has been defined, there is a large conceptual gap between this *in vitro* phenomenon and the pathway that leads to *in vivo* development of lymphadenopathy and autoimmunity. A striking abnormality in *lpr* mice is the excessive proliferation of CD4^+^ and CD8^+^ T cells, and more so of the double-negative TCR^+^CD4^−^CD8^−^B220^+^ T cells. The basis of *lpr* T cell hyperproliferation remains elusive, as it cannot be explained by Fas-deficient apoptosis. T cell-directed p21 overexpression reduces hyperactivation/hyperproliferation of all *lpr* T cell subtypes and lymphadenopathy in *lpr* mice. p21 controls expansion of repeatedly stimulated T cells without affecting apoptosis. These results confirm a direct link between hyperactivation/hyperproliferation, autoreactivity, and lymphadenopathy in *lpr* mice and, with earlier studies, suggest that Fas apoptosis-independent pathways control *lpr* T cell hyperproliferation. *lpr* T cell hyperproliferation could be an indirect result of the defective apoptosis of repeatedly stimulated *lpr* T cells. Nonetheless, in this perspective, we argue for an alternative setting, in which lack of Fas would directly cause *lpr* T cell hyperactivation/hyperproliferation *in vivo*. We propose that Fas/Fas ligand (FasL) acts as an activation inhibitor of recurrently stimulated T cells, and that its disruption causes overexpansion of T cells in *lpr* mice. Research to define the underlying mechanism of this Fas/FasL effect could resolve the phenotype of *lpr* mice and lead to therapeutics for related human syndromes.

## Fas-Dependent Apoptosis of T Cells

Homeostasis regulates total lymphocyte number by balancing cell growth and death. Programmed cell death, referred to as apoptosis, eliminates activated or autoreactive lymphocytes and is thus important in immune system homeostasis. Interaction of Fas (CD95), a member of the tumor necrosis factor receptor family, with its ligand [(Fas ligand (FasL)] after *in vitro* recurrent T cell receptor (TCR) triggering, induces apoptosis *via* activation-induced cell death (AICD). Initiation of AICD requires IL-2 exposure prior to secondary TCR stimulation (1). Fas/FasL interaction recruits the adaptor protein Fas-associated death domain (FADD), which activates caspase-8 and initiates the apoptotic cascade. T cell apoptosis is considered central to lymphocyte homeostasis and tolerance induction (1).

## Phenotype and Characteristics of Fas-Deficient Mice

*lpr* (lymphoproliferation spontaneous mutation) mice deficient in Fas show defective AICD of restimulated T cells *in vitro*. *lpr* mice present lymphadenopathy due to double-negative (DN) T cell (TCR^+^CD4^−^CD8^−^B220^+^) hyperproliferation and accumulation. They also develop lupus-like autoimmune disease, probably due to CD4^+^ T cell hyperactivation. The severity of these symptoms depends on genetic background. B6/*lpr* mice develop anti-DNA antibodies and mild, non-lethal glomerulonephritis, whereas *lpr* mice on the MRL background (MRL/*lpr*) show more pronounced autoimmune manifestations, thereby leading to kidney failure and death (1); this severe phenotype is a combined result of the autoimmune-prone background and the *lpr* mutation. *lpr* mice of both backgrounds develop severe lymphadenopathy and splenomegaly.

The hyperproliferative T cell phenotype of *lpr* mice is also observed in patients with autoimmune lymphoproliferative syndrome (ALPS) (2–4), an autoimmune disease also characterized by defective Fas/FasL signaling. ALPS patients are classified by distinct disease types, depending on the underlying genetic defect (5). The main characteristics of this syndrome are DN T cell accumulation and hyperproliferation, lymphadenopathy development, autoimmune manifestations, and increased risk of lymphomas.

## The Controversy Over Fas-Dependent T Cell Apoptosis *In Vivo*

Although lack of Fas-triggered apoptosis in *in vitro*-activated T cells of *lpr* mice was initially suggested to be a direct cause of lymphadenopathy and lupus-like disease (6), the etiology of these symptoms remains enigmatic. While Fas-dependent apoptosis has been clearly established and extensively studied in IL-2-exposed and -restimulated T cells, *in vivo* Fas/FasL-induced apoptosis and T cell elimination have been questioned. All evidence for the Fas/FasL apoptosis pathway is based essentially on *in vitro* experiments of recurrent T cell activation, and the *in vivo* role of this system thus remains ill defined. For example, peptide-induced deletion of T cells in TCR transgenic mice was reported to be Fas dependent in one model, whereas it was Fas independent in other settings (7, 8). Superantigen-induced T cell deletion is dependent on Fas in some systems but not in others, and it was concluded that dissimilar experimental conditions might alter Fas effectiveness (9). Peripheral DN T cells appear to depend on Fas for superantigen-induced apoptosis (10). In addition, defective AICD was not identified following *in vivo* T cell activation in mice in which Fas was specifically deleted in T cells (11). *In vivo* Fas-dependent apoptosis is thus not clearly defined in *in vivo* systems (1), and the debate continues as to how Fas deficiency leads to DN T cell accumulation and lymphadenopathy development.

This debate was further fueled by research directed toward defining whether inactivation of components of the Fas/FasL apoptosis system could reproduce the disease-prone *lpr* phenotype. Transgenic mice were produced that overexpress the caspase-8 inhibitor CrmA in T cells, and AICD of these cells was efficiently inhibited *in vitro* (12, 13). The CrmA transgenic mice, nonetheless, showed no T cell abnormalities *in vivo*, no lymphadenopathy, and no predisposition to autoimmunity (12, 13); it was thus suggested that in addition to its role in apoptosis, Fas has other functions in the control of *in vivo* T cell homeostasis (12). In other studies, mice were rendered deficient in the major apoptotic regulators caspase-8 or FADD, but again, the *lpr in vivo* symptoms were not replicated (14, 15); instead, both caspase-8 and FADD were shown to be necessary for normal T cell proliferation. These studies predicted a possible Fas association not only to apoptosis but also to T cell proliferation, and it was proposed that the defective *in vitro* apoptosis of restimulated T cells might not be related to the *lpr* phenotype (16).

## Hyperproliferation of all *lpr* Mouse T Cell Subsets

A critical characteristic of the *lpr* phenotype is extensive T cell hyperproliferation, which is not explained by the defective Fas/FasL apoptotic system. DN CD4^+^ and CD8^+^ T cells hyperproliferate *in vivo* (17–19) and memory T cells (CD44^high^/CD62L^low^) expand massively in lymphoid organs. DN *lpr* T cells were initially considered inert or anergic cells that accumulate in lymph nodes (20, 21). This does not concur with their hyperproliferative state or with the fact that they secrete large amounts of inflammatory cytokines such as IFN-γ and IL-17 and can be pathogenic (22–24). Furthermore, IL-23 is essential for lymphadenopathy and DN T cell expansion, as disrupted signaling by this cytokine leads to lower numbers of DN T cells (22). The active state of DN cells is supported by recent findings that normal programmed cell death protein 1-expressing DN cells are pro-inflammatory and respond to self-antigens (25, 26) and by their association with human lupus (27, 28).

**Figure 1:**
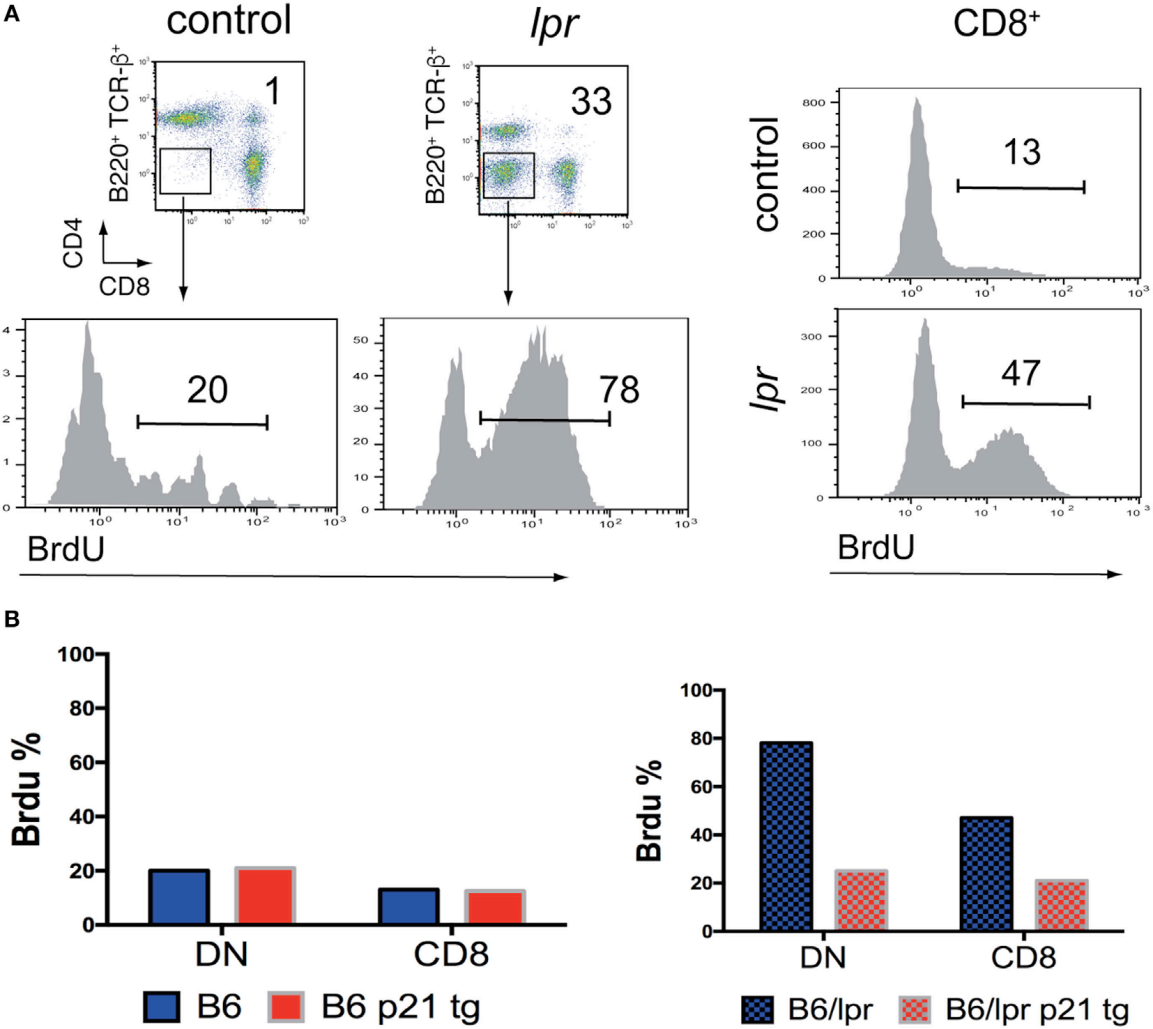
***In vivo* hyperproliferation of *lpr* T cells is moderated by p21**. **(A)** Increased proliferation of B6/*lpr* double-negative (DN) and CD8^+^ T cells compared to control B6 cells. Lymph node cells were obtained from mice, which received BrdU in the drinking water for 8 days. FACS analysis was used to identify cell types and percentage of proliferating BrdU^hi^ T cells. **(B)** Effect of a T cell-directed p21 transgene on the proliferation levels of DN and CD8^+^ T cells from lymph nodes of B6 and B6/*lpr* mice, detected by BrdU incorporation as in A. The p21 transgene did not affect the levels of low proliferating DN and CD8^+^ B6 T cells, while it reduced by more than 50% the BrdU uptake by the corresponding B6/*lpr* cells.

Lymph node *lpr* DN cells proliferate *in vivo* at much higher rates than CD8^+^ T cells (Figure 1A). *lpr* DN T cells might originate from activated single-positive T cells that have lost the CD8 receptor (29); however, they have a much higher proliferation rate than CD8^+^ cells. Alternatively, it was recently argued that *lpr* DN cells correspond to normal DN T cells with inherently increased proliferation than CD4^+^ or CD8^+^ T cells (10, 28). Regardless of their origin, DN cells proliferate at much higher rates and their homeostasis might be Fas/FasL pathway dependent (10, 28).

T cell hyperproliferation is also observed in patients with ALPS (2, 4), an autoimmune disease characterized by defective Fas/FasL signaling or by other genetic defects associated with the Fas/FasL pathway. The major features of this syndrome are DN T cell accumulation, lymphadenopathy development, autoimmunity, and increased lymphoma risk (5).

To explain the lymphoproliferative and autoimmune *lpr* phenotype, we suggested that Fas deficiency also leads to T cell proliferation abnormalities (30), a view previously proposed based on studies of ALPS patients (4). Indeed, treatment of ALPS patients includes drugs that target the activation/proliferation aspects of accumulating T cells, such as mycophenolate mofetil and sirolimus (rapamycin) (31).

While Fas functions other than apoptosis are well established (32), and *lpr* T cell hyperproliferation appears to be essential for development of the *lpr* phenotype, Fas-deficient apoptosis is still considered the prevailing cause of lymphadenopathy and autoimmunity development in *lpr* mice.

## Reduction of T Cell Hyperproliferation Regulates Lymphadenopathy and Lupus Development in *lpr* Mice

To date, no direct evidence has correlated the defective *in vitro* apoptosis of *lpr* T cells with *in vivo* T cell hyperproliferation, lymphadenopathy, and autoimmune disease in *lpr* mice. The magnitude of hyperproliferation of all T cell subsets in *lpr* mice and ALPS patients suggests that this abnormality could be relevant in the development of lupus and lymphadenopathy.

Recent research from our laboratory addressed this point, demonstrating that reduction of *lpr* T cell hyperproliferation effectively suppressed lymphadenopathy and autoimmune disease development in these mice (30). In a previous study, T cell blasts from ALPS patients with higher responses than controls showed lower p21 expression (4). We therefore overexpressed the cell cycle inhibitor p21 in B6/*lpr* and MRL/*lpr* mice in a T cell-specific manner. p21 is an established negative regulator of repeatedly stimulated but not of naïve T cells and regulates CD4^+^ effector/memory T cell expansion (33, 34).

In general, overexpressed p21 diminished *in vivo* hyperproliferation of DN cells to almost normal levels and greatly reduced lymphadenopathy in *lpr* mice (Figure 1B). p21 also decreased CD4^+^ and CD8^+^ *lpr* T cell hyperproliferation and autoimmune disease development. Importantly, p21 reduced the activated phenotype of these cells as well as that of DN cells and downregulated their potential to produce IFN-γ and IL-17. p21 also detained *lpr* T cell hyperactivity, which suggests that p21 regulates activation pathways. In fact, p21 suppresses macrophage activation through the NF-κB activation pathway (35, 36) and inhibits inflammatory cytokine production (35–38) in a cell cycle-independent manner.

Overexpression of p21 had no effect on the *in vivo* proliferation/activation of normal background T cells (CD4^+^, CD8^+^, and DN) (Figure 1B), or their effector/memory T cell expansion. Therefore, p21 effects on *lpr* hyperproliferation and lymphadenopathy are not caused by p21 influence on essential normal T cell functions. It appears that lack of Fas interferes with the regulation of activation and proliferation, which is then adjusted by overexpressed p21. Recurrent *in vitro* activation of p21-overexpressing wt and *lpr* T cells showed no effect on apoptosis induction in wt T cells and did not restore the defective apoptosis of *lpr* T cells. This finding indicates that p21 reduced the *lpr* lymphoproliferation without interfering with apoptosis pathways.

p21 thus reduced *lpr* T cell hyperproliferation, lymphadenopathy, and autoimmune disease development in *lpr* mice, showing that this hyperproliferation is critical for lymphadenopathy and lupus-like disease development.

## Etiology of *In Vivo lpr* T Cell Proliferation

Whereas T cell hyperproliferation is central to the development of lymphadenopathy and autoimmunity in *lpr* mice, the cause of the hyperproliferation/hyperactivation of all *lpr* T cell subsets remains unclear (17–19). Suppression of *in vivo lpr* T cell hyperproliferation by overexpressed p21 indicated that Fas deficiency alters regulation of the proliferation machinery in T cells, rendering *lpr* T cells prone to hyperactivation and hyperproliferation. This view is further supported by the finding that p21 overexpression has no effect on proliferation/activation of wt T cells with intact Fas/FasL signaling [Figure 1B; Ref. (30)].

The question that arises at this point is whether *lpr* T cell hyperproliferation is an indirect consequence of the Fas deficiency or whether Fas has a direct apoptosis-independent antiproliferative effect on T cells. Here, we will explore these two ideas.

### The Possibility That Deficient Fas/FasL Apoptosis Drives *lpr* T Cell Hyperactivation and Hyperproliferation

**Figure 2:**
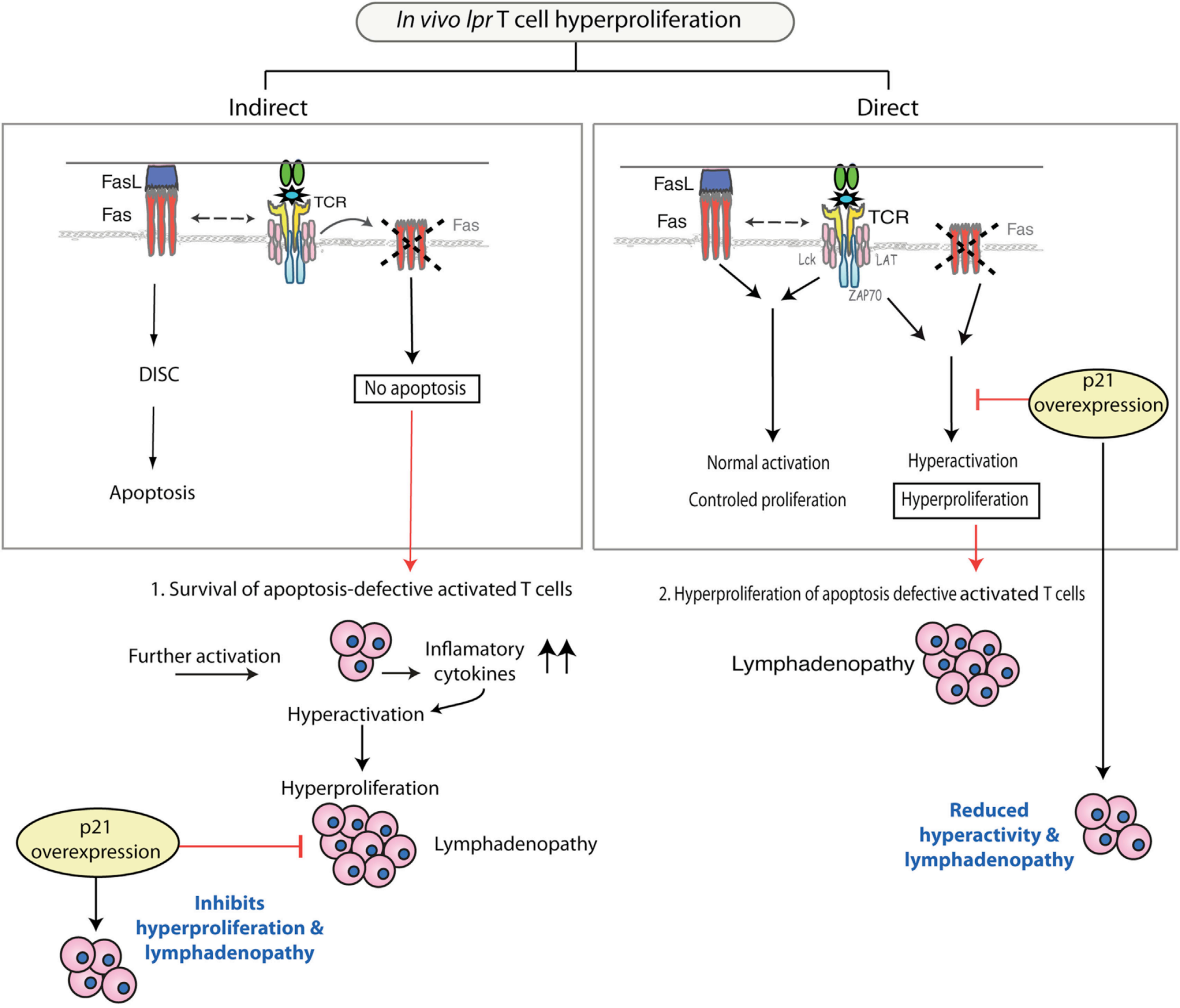
**Pathways that could produce the *in vivo* hyperproliferation of *lpr* T cells**. Left: Fas-deficient T cells that fail to undergo apoptosis might acquire an indirect increased capacity for proliferation, enhanced by inflammatory cytokines that create a feedback loop and result in hyperproliferating *lpr* T cells. p21 overexpression inhibits hyperproliferation of these cells, which reduces lymphadenopathy. Right: Fas-deficient T cells show a hyperproliferative phenotype due to lack of Fas, which after interacting with Fas ligand (FasL), has a direct regulatory effect on activation/proliferation. These hyperactivated *lpr* T cells accumulate in lymph nodes. p21 overexpression reduces hyperactivation/proliferation of *lpr* T cells, and lymphadenopathy development is minimal.

To date, the *in vivo* proapoptotic role of Fas on T cells has been tested in various experimental models, but as mentioned above, there is no conclusive evidence to support an *in vivo* apoptotic effect for Fas. In the case that Fas would play such a role, however, it could be envisaged that activated apoptosis-deficient *lpr* T cells would accumulate (Figure 2, left). After reactivation, this population would secrete inflammatory cytokines such as IFN-γ, IL-17, and IL-23, and through a feedback mechanism could generate an environment that drives *lpr* T cell hyperactivation and hyperproliferation. Overexpressed p21 could reduce this *lpr* T cell hyperproliferation. Nonetheless, as the *in vivo* proapoptotic Fas/FasL effect remains unclear, the view that *lpr* T cell hyperproliferation is an indirect effect of deficient apoptosis remains hypothetical.

### The Fas/FasL System As an Attenuator of T Cell Activation and Proliferation

A number of *in vitro* studies support a role for Fas/FasL in the regulation of T cell activation and proliferation. While in naïve T cell activation, Fas acts as a costimulatory molecule, in preactivated cells, the FasL/FasL interaction has the opposite effect and appears to inhibit T cell activation. Suggestive evidence for an *in vivo* Fas/FasL role as a negative regulator of T cell activation is derived from *lpr* mice that overexpress p21 in T cells, as high p21 levels controlled *lpr* T cell activation and proliferation as well as autoimmunity and lymphadenopathy. Here, we discuss and evaluate this facet of the Fas/FasL system.

#### The Dual Role of Fas in Regulating Naïve T Cell Activation

Experimental evidence from primary activation of Fas- and FasL-deficient T cells showed a role for Fas as a costimulatory molecule in naïve T cell activation (39), and other studies analyzed the mechanism of this costimulatory Fas effect (40).

In contrast to the role of Fas/FasL as enhancer of T cell activation, high-dose FasL inhibits TCR-dependent activation of human peripheral cells or naïve T cells (41, 42). It appears that FasL has an inhibitory effect on T cell activation only at high concentrations, while at low concentration, it acts as a costimulatory factor. The mechanisms of FasL interference with T cell activation and proliferation are reviewed by Paulsen and Jansen and Brint et al. (40, 43).

As large amounts of FasL were used in experiments that showed the inhibitory effect of Fas/FasL on T cell activation, it might be argued that the results imply a non-physiological effect of the Fas/FasL system. These findings nonetheless provide a basis for the hypothesis that Fas/FasL interactions inhibit *in vivo* activation and proliferation of T cells in certain conditions.

#### Fas Triggering Reduces the Response of Activated T Cells

Experiments using FasL treatment indicate a role for Fas/FasL signaling in naïve T cell activation and proliferation, as commented above. Previously activated T cells are also sensitive to Fas signaling, which reduces their activation and expansion potential. Signaling through Fas elicits antiproliferative effects, a finding first reported by the Tsokos laboratory in 1995 (44). In that study, Fas cross-linking inhibited subsequent anti-CD3 activation of human IL-2-dependent T cells. The same group confirmed this concept using Jurkat T cells (45). In a later study (4), Fas binding of human T cell blasts reduced their proliferation with a concomitant increase in p21, a finding that directly associates p21 with the antiproliferative mechanism of the Fas/FasL system. These studies reinforce the view that the Fas/FasL system attenuates proliferation, although Fas cross-linking *in vitro* could entail questions regarding the physiological relevance of these data.

#### Defective Fas/FasL Signaling Leads to *lpr* T Cell Hyperactivation and Is Counteracted by p21 Overexpression

One of the major autoimmune characteristics of *lpr* mice is hyperactivation of the immune response and specifically, of T cells. Use of *lpr-cREL^−/−^* mice (46) showed that lupus-like disease depends on immune response activation by c-REL, a component of the NF-κB system, whereas absence of NF-κB1 (p65) in *lpr* mice led to reduced lymphadenopathy. These data argue that hyperactivation is essential for *lpr* T cell hyperproliferation and corroborate previous results showing that reduction in DN cell activity leads to reduced lymphadenopathy (17).

Here, we propose a potential Fas/FasL role in negative regulation of T cells repeatedly stimulated by autoantigen that could control autoimmunity independently of the classical role of this system in *in vitro* T cell apoptosis. This possibility is reinforced by the abovementioned studies in which FasL treatment of T cells reduced their activation and proliferation capacity. This view would concur with the development of hyperactivated T cells, whose hyperproliferation leads to their accumulation in the lymphoid organs of *lpr* mice.

In the case of p21 overexpression in *lpr* mouse T cells, it can be considered that p21 directly suppresses the deregulated activation of *lpr* T cells. This would adjust the hyperproliferative state of *lpr* T cells and inhibit development of autoimmunity and lymphadenopathy (Figure 2, right). This view of a direct p21 effect on *lpr* T cell activity is supported by *in vivo* experiments in which p21 overexpression by *lpr* T cells reduced activation marker expression and IFN-γ production, which finally reduced proliferative capacity (30). In *in vitro* experiments, overexpressed p21 was detected shortly after secondary stimulation and coincided with reduction of T cell activation. These results link p21 to regulation of T cell activation, as p21 had no effect on Fas/FasL apoptosis. p21 overexpression leads to early reduction of extracellular signal-regulated kinase activity (30), a finding that further supports a role for p21 in controlling recurrent *lpr* T cell activation. This p21 function is further reinforced by studies showing that p21 attenuates NF-κB-dependent macrophage activation (35, 36). Unpublished data from our laboratory indicate a role for normally expressed p21 in T cell activation after secondary stimulation. Evidence that p21 regulates T cell activation after secondary apoptosis would further strengthen the view that lack of Fas/FasL signaling leads directly to T cell hyperactivation.

By introducing the idea that defective Fas/FasL signaling generates T cell hyperactivation/hyperproliferation in *lpr* mice independent of the apoptotic defect, we supply a model that explains autoimmunity and lymphadenopathy in these mice. It is our hope that this perspective will prompt research that clarifies the etiology of the *lpr* mouse phenotype and explains how Fas/FasL interaction modulates T cell activation and proliferation.

## Emerging Views

Although Fas is considered a central molecule in T cell apoptosis, and the *in vitro* apoptosis effects of the Fas/FasL pathway have been studied extensively, the influence of this system on the *lpr* mouse phenotype remains unclear. With the exception of some recent studies, research in T cells has shifted mainly to other molecules that control homeostasis. Most of our understanding of Fas/FasL is based on analyses in other tissues and systems, and the role of Fas in lymphadenopathy and autoimmunity has remained undefined. We thus consider it imperative to determine how Fas contributes to T cell-driven autoimmunity, since MRL-*lpr* lupus-like disease is a prevalent model for autoimmunity studies. In this perspective, we explain how p21-based reduction in *lpr* T cell hyperactivation led us to link *lpr* T cell hyperactivation to lymphadenopathy and lupus development in *lpr* mice and ALPS patients. This hypothesis evokes the exciting possibility that impaired Fas/FasL signaling could control *lpr* T cell activation and proliferation directly. Further research is needed to address this prospect and draw to a close the debate as to how defective Fas apoptosis leads to the *lpr* phenotype. Perhaps, recent mechanistic evidence showing that Fas drives distinct activities (47, 48) will help to define the diverse Fas pathways involved in T cell function.

## Ethics Statement

All animal experiments and protocols were designed in compliance with European Union directives and guidelines and were approved by the Centro Nacional de Biotecnología (CNB-CSIC) Ethics Committee.

## Author Contributions

DB conceived and wrote the manuscript; RS composed the final versions of the figures and organized the argument and the manuscript; LD and CV-M contributed to the content and composition of the figures; CM-A contributed to the intellectual content of the manuscript. All the authors read and approved the final version of the manuscript.

## Conflict of Interest Statement

The authors declare that the research was conducted in the absence of any commercial or financial relationships that could be construed as a potential conflict of interest.
